# Association between mood disorders and frequent emergency department use: a cross-sectional study

**DOI:** 10.1007/s43678-021-00204-w

**Published:** 2021-10-20

**Authors:** Christophe A. Fehlmann, Marcel Miron-Celis, Yue Chen, Jeffrey Perry, Debra Eagles

**Affiliations:** 1 Ottawa Hospital Research Institute, Ottawa, ON Canada; 2 School of Epidemiology and Public Health, University of Ottawa, Ottawa, ON Canada; 3 Department of Anaesthesiology, Clinical Pharmacology, Intensive Care and Emergency Medicine, Geneva University Hospitals, Geneva, Switzerland; 4 Department of Emergency Medicine, University of Ottawa, Ottawa, ON Canada

**Keywords:** Emergency department, Frequent, Psychiatric, Mood disorders, Geriatric

## Abstract

**Objectives:**

Frequent emergency department (ED) use is a growing problem that is associated with poor patient outcomes and increased health care costs. Our objective was to analyze the association between mood disorders and the incidence of frequent ED use.

**Methods:**

We used the Canadian Community Health Survey conducted by Statistics Canada, 2015–2016. Mood disorder was defined as depression, bipolar disorder, mania, or dysthymia. Frequent ED use was defined as 4 or more visits in the year preceding the interview. Multivariable log-binomial regression models were used to determine the associations between mood disorders and frequent ED use.

**Results:**

Among the 99,009 participants, 8.4% had mood disorders, 80.3% were younger than 65, and 2.2% were frequent ED users. Mood disorders were significantly associated with the 1-year cumulative incidence of frequent ED use (RR = 2.5, 95% CI 2.2–2.7), after adjusting for several potential confounders.

**Conclusions:**

This national survey showed that people with a mood disorder had a three-fold risk of frequent ED use, compared to people without mood disorder. These results can inform the development of policies and targeted interventions aimed at identifying and supporting ED patients with mood disorder.

## Résumé

**Objectifs:**

L'utilisation fréquente des services d'urgence est un problème croissant qui est associé à de mauvais résultats pour les patients et à une augmentation des coûts des soins de santé. Notre objectif était d'analyser l'association entre les troubles de l'humeur et l'incidence de l'utilisation fréquente des urgences.

**Méthodes:**

Nous avons utilisé l'Enquête sur la santé dans les collectivités canadiennes menée par Statistique Canada en 2015-2016. Le trouble de l'humeur est défini comme une dépression, un trouble bipolaire, une manie ou une dysthymie. L'utilisation fréquente des urgences est définie comme 4 visites ou plus dans l'année précédant l'entretien. Des modèles de régression log-binomiale multivariés ont été utilisés pour déterminer les associations entre les troubles de l'humeur et l'utilisation fréquente des services d'urgence.

**Résultats:**

Parmi les 99 009 participants, 8,4 % souffraient de troubles de l'humeur, 80,3 % avaient moins de 65 ans et 2,2 % utilisaient fréquemment les services d'urgence. Les troubles de l'humeur étaient significativement associés à l'incidence cumulative sur un an d'utilisation fréquente des urgences (RR = 2,5, IC à 95 % 2,2-2,7), après ajustement pour plusieurs facteurs de confusion potentiels.

**Conclusions:**

Cette enquête nationale montre que les personnes souffrant d'un trouble de l'humeur ont un risque trois fois plus élevé de recourir fréquemment aux services d'urgence, par rapport aux personnes sans trouble de l'humeur. Ces résultats peuvent contribuer au développement de politiques et d'interventions ciblées visant à identifier et à soutenir les patients souffrant de troubles de l'humeur aux urgences.

**Clinicians’ capsule**

***What is known about the topic?***

Risk factors for frequent emergency department (ED) use include age and mental health conditions.

***What did this study ask?***

What is the association between mood disorders and being a frequent ED user?

***What did this study find?***

Our study suggests that there is positive association between mood disorders and frequent ED use.

***Why does this study matter to clinicians?***

This study should encourage the development of policies and targeted interventions for patients with mood disorders to decrease the burden associated with frequent ED use.

## Introduction

Frequent emergency department (ED) users, usually defined as patients with four or more emergency department visits per year, are a critical issue in emergency medicine. They represent between 4 and 16% of all ED users and account for 15% to almost 50% of all ED visits. Frequent ED use is associated with increased hospital admission and mortality [1]. The number of frequent ED users is rising, with a 66% increase over 10 years. This has significant implications for patients and the healthcare system contributing to the overcrowding of EDs and increased resource utilization.

Known risk factors for frequent ED use include female sex, young age, low socioeconomic status, low education level, substance use, fair or poor self-reported health, increased number of chronic conditions and mental health conditions (depression, anxiety). Mental health conditions are especially important because the proportion of ED visits due to mental illness increased by almost 30 percent, from 48.7 per 1000 ED visits in 1992 to 62.5 in 2001 [2]. Moreover, patients with depression or anxiety have a higher risk of hospitalization for non-psychiatric conditions, by affecting self-management and treatment adherence [3].

Most of the studies on frequent ED users were performed outside Canada. A recent study identified patients’ characteristics associated with frequent ED use in Alberta and Ontario [4]. However, this study was based on administrative data, without health-related variables. A better understanding of factors associated with frequent ED use in our country is needed to develop targeted interventions to improve care for this population and to increase efficient health care utilization.

The objective of this study was therefore to investigate the association between mood disorders and frequent emergency department use.

## Methods

This report follows the STROBE Statement guidelines for reporting cross-sectional studies.

### Study design, setting, and participants

This study analyzed data from the Canadian Community Health Survey (CCHS)—Mental Health collected by Statistics Canada (Ottawa, Ontario) in 2015–2016. The CCHS is a cross-sectional survey and collects information on various dimensions of health for the Canadian population. For this analysis, we included adult participants aged 18 or over and excluded those with missing values for mood disorders or ED visits.

### Variables

Frequent ED use was defined as 4 or more visits to the ED in the past 12 months, the most common cut-off. Mood disorder was defined as positive if participants answered “Yes” to the question “Do you have a mood disorder such as depression, bipolar disorder, mania or dysthymia?”. Potential confounders were age, sex, living arrangement, marital status, educational attainment, immigrant status, alcohol consumption, smoking status, having a regular health care provider, presence of a chronic health condition and total household income.

### Statistical analysis

As this was a study based on a survey already realized, no formal sample size calculation was computed. We measured the 1-year cumulative incidence of frequent ED use according to mood disorders, age, and other study variables. A Chi-square test was used for group comparison. Missing data were recoded as “unknown”. Log binomial regression model was used to evaluate the association between the mood disorders and frequent ED use, before and after adjusting for confounders, and crude and adjusted risk ratios (RR) were calculated. Covariates were chosen a priori, based on previous knowledge. We performed a sensitivity analysis to test different cut-offs for the definition of frequent ED use (3 and 5 visits a year). For all tests, a two-sided *p* value below 0.05 was considered significant. Finally, the CCHS used a complex survey design, with stratification, clustering, and unequal selection probabilities for sampling. This was taken into consideration by using sampling weight and average design effect. Statistical analysis was performed using STATA version 17 (Stata Corporation, College Station, TX, USA).

## Results

A total of 100,679 adults participated in the CCHS 2015–16. We excluded 1670 (1.7%) records for missing information (195 for mood disorder, 1475 for ED use), thus 99,009 people were included in the analysis. Among them, 9424 (8.4%) had a mood disorder.

Overall, the 1-year cumulative incidence of frequent ED use was 2.2%. Frequent ED use was more common in people with mood disorders than in people without (6.7% versus 1.8%, *p* < 0.001). Table 1 shows the incidence of frequent ED use among different risk factors, stratified by mood disorder.

**Table 1:** One-year cumulative incidence of frequent emergency department use according potential risk factors, stratified by mood disorders (*N* = 99,009)

	No mood disorder			Mood disorder		
	Total	Frequent ED users	%^a^	Total	Frequent ED users	%^a^
Age (years)						
18–34	19,555	608	2.2	2163	227	8.6
35–54	26,164	540	1.5	3260	246	5.6
55–64	16,626	300	1.4	2057	114	5.0
65–74	15,946	318	1.4	1298	79	7.0
75 and over	11,294	351	2.9	646	45	10.9
Sex						
Male	42,271	929	1.7	3169	200	5.0
Female	47,314	1188	1.9	6255	511	7.1
Marital status						
Married/Common-law	50,916	1058	1.6	3864	273	5.7
Widowed/divorced/separated	18,796	488	2.3	2636	180	8.1
Single	19,586	561	2.0	2901	254	7.4
Unknown	287	10	1.9	23	4	16.9
Living arrangement						
Living alone	25,787	614	1.9	3601	254	6.2
Living with others	63,330	1479	1.7	5769	449	6.9
Unknown	468	24	4.3	54	8	5.0
Highest level of education						
Less than secondary school	14,144	458	2.6	1742	166	10.3
Secondary school	20,244	529	2.1	2312	215	7.9
Post-secondary	54,057	1104	1.5	5253	324	5.3
Unknown	1140	26	1.7	117	6	3.6
Total household income						
Low	7935	315	3.1	2007	216	8.7
Medium	32,051	909	2.4	3647	281	7.8
High	49,486	888	1.4	3756	214	5.3
Unknown	113	5	6.0	14	0	0.0
Immigrant status						
Non-immigrant	72,962	1885	2.1	8270	641	7.0
Immigrant	14,873	184	1.0	1005	57	5.0
Unknown	1750	48	2.2	149	13	6.9
Alcohol consumption						
Regular drinker	56,601	1073	1.4	4989	327	5.6
Occasional drinker	14,755	466	2.6	1983	172	8.1
Did not drink in the last 12 months	17,780	569	2.3	2404	210	8.1
Unknown	449	9	1.5	48	2	9.4
Smoking status						
Current smoker	16,406	546	2.9	3129	297	7.7
Former smoker	27,966	703	1.8	2839	190	6.3
Abstainer/experimental smoker	44,833	858	1.4	3422	220	6.2
Unknown	380	10	3.3	34	4	7.9
Regular health care provider						
Yes	75,018	1787	1.8	8395	634	6.8
No	14,471	329	1.6	1021	77	6.2
Unknown	96	1	2.2	8	0	0.0
Presence of a chronic health condition						
No	57,671	1031	1.4	4658	283	5.1
Yes	31,194	1086	2.8	4776	428	8.9

^a^One-year cumulative incidence estimates were weighted to the general population

Mood disorder was significantly associated with frequent ED use, with an unadjusted RR of 3.8 (95% CI 3.4–4.1). People with mood disorder had a 2.5-fold higher risk to be frequent ED users (95% CI 2.2–2.7), compared to people without mood disorder after adjustment for age, sex, marital status, living status, education level, total household income, immigrant status, alcohol consumption, smoking status, and presence of chronic health conditions. This association was persistent in our sensitivity analyses.

## Discussion

This large national study, based on data collected through the Canadian Community Health Survey, showed that mood disorder was associated with frequent ED use.

Our finding that patients with mood disorders were 2.5 times more likely to be frequent ED users is consistent with previous studies. In a large-scale US case–control study, Niedzwiecki et al. showed that mental health diagnosis during an ED visit, primary discharge diagnosis related to mental health, and more severe mental health diagnosis were all associated with more ED visits in the following year [5]. However, their results were based on discharge diagnoses rather than mental illness as a comorbidity. In our study, we focussed on mental illness as a comorbidity, to give a more global picture. In the same goal and based on in-person interviews, Mehl-Madrona demonstrated that 93% of frequent ED users had a psychiatric comorbidity, compared to 50% of matched non-frequent users [6]. However, as the number of patients was small, no adjustment was possible. Moreover, about half of the identified frequent users refused to participate, which may have resulted in selection bias. Finally, Hunt et al., based on a similar survey, showed that poor mental health was associated with a higher likelihood of frequent ED use, with an adjusted odds ratio of 1.70 [7]. The higher risk ratio we found in our analysis could be explained by the use of different exposure measurements (i.e. a diagnosis of mood disorder rather than a score of mental health).

This study has several noteworthy strengths. Firstly, our results are based on a large sample size that allows us to observe even small differences and control for many variables, increasing the internal validity of the results. Secondly, this was a national survey with a robust sampling strategy. Then, to define frequent ED use, we adopted the most commonly used definition but did a sensitivity analysis using other cut-offs that confirmed our results.

Some limitations should be mentioned. A cross-sectional study does not allow conclusions to be drawn regarding causality, as such, it is possible that frequent ED use could also be a cause of mood disorders or could increase mood disorders diagnosis. A second limitation is the survey design, as most of the variables, were self-reported. This could result in misclassification and could bias our results toward or away from the null and affect the validity of our results. The same limitation applies to confounders such as socially undesirable behaviours, where patients could have underreported certain behaviours. Another limitation is the risk of residual confounding, which can affect the internal validity of our conclusion, especially if an important confounder was omitted. Finally, the generalizability of our results should be restricted to similar populations, especially in terms of health system organization and health conditions.

This study has some potential but important clinical implications. Identifying patients with a high risk of frequent ED use could help improve their care. For mood disorders, a screening tool as proposed by Chang et al. can be used in the identification process [8]. Patients who are at high risk for frequent ED use could then be targeted for both ED and non–ED based interventions, such as case management programs [9]. Such interventions seem to be effective in decreasing ED visits. ED patients with mood disorder symptoms are likely to have complex psychiatric, medical and social histories that may need special ED-initiated interventions [10].

Some research perspectives are also to be noted. Although this study demonstrated an increased risk of frequent ED use among the psychiatric population, the reason for the ED visit was not documented. Frequent ED users likely attend the ED for psychiatric and non-psychiatric-related issues. A better understanding of the reason for ED attendance can be used to tailor specific interventions. Moreover, interventions targeting frequent ED users with mood disorders are scarce. Developing and testing such interventions could improve care for this vulnerable population.

In summary, this large, national survey showed that people with a mood disorder had a 2.5-fold risk to be frequent ED users, compared to people without mood disorder. These results can inform the development of policies and targeted interventions aimed at identifying and supporting ED patients with mood disorder, in an effort to mitigate the negative patient and healthcare system sequelae associated with frequent ED use.

## Data Availability

The data that support the findings of this study have restrictions and so are not publicly available. Data are, however, available from the authors upon reasonable request.
